# Effect on Body Composition of a Meal-Replacement Progression Diet in Patients 1 Month after Bariatric Surgery

**DOI:** 10.3390/nu16010106

**Published:** 2023-12-28

**Authors:** Juan J. López-Gómez, Beatriz Ramos-Bachiller, David Primo-Martín, Alicia Calleja-Fernández, Olatz Izaola-Jauregui, Rebeca Jiménez-Sahagún, Jaime González-Gutiérrez, Eva López Andrés, Pilar Pinto-Fuentes, David Pacheco-Sánchez, Daniel A. De Luis-Román

**Affiliations:** 1 Endocrinology and Nutrition Department, Clinic Universitary Hospital of Valladolid, 47003 Valladolid, Spain; 2 Investigation Centre Endocrinology and Nutrition, Faculty of Medicine, University of Valladolid, 47003 Valladolid, Spain; 3 General Surgery Department, Rio Hortega Universitary Hospital, 47012 Valladolid, Spain

**Keywords:** bariatric surgery, oral nutritional supplement, hyperproteic diet, sleeve gastrectomy

## Abstract

Background: Progression diets after bariatric surgery (BS) are restricted in calories and protein, and they may induce a worsening of body composition. The aim of this study was to evaluate the effect of a modified diet with an oral nutritional supplement that is hyperproteic and normocaloric over the body composition. Methods: A two-arm ambispective observational cohort study was designed. Forty-four patients who underwent sleeve gastrectomy were included in the study. Thirty patients received a progression diet with a normocaloric, hyperproteic oral nutritional supplement during the first two weeks after surgery (820 kcal, 65.5 g protein). They were compared with a historical cohort of 14 patients treated with a standard progression diet (220 kcal, 11.5 g protein). Anthropometric and body composition (using electrical bioimpedanciometry) data were analyzed before BS and 1 month after the surgery. Results: The mean age was 47.35(10.22) years; 75% were women, and the average presurgical body mass index (BMI) was 45.98(6.13) kg/m^2^, with no differences between both arms of intervention. One month after surgery, no differences in the percentage of excess weight loss (%PEWL) were observed between patients in the high-protein-diet group (HP) and low-protein-diet group (LP) (HP: 21.86 (12.60)%; LP: 18.10 (13.49)%; *p* = 0.38). A lower loss of appendicular skeletal muscle mass index was observed in the HP (HP: −5.70 (8.79)%; LP: −10.54 (6.29)%; *p* < 0.05) and fat-free mass index (HP: 3.86 (8.50)%; LP:−9.44 (5.75)%; *p* = 0.03), while a higher loss of fat mass was observed in the HP (HP: −14.22 (10.09)%; LP: −5.26 (11.08)%; *p* < 0.01). Conclusions: In patients undergoing gastric sleeve surgery, the addition of a normocaloric, hyperproteic formula managed to slow down the loss of muscle mass and increase the loss of fat mass with no differences on total weight loss.

## 1. Introduction

The World Health Organization (WHO) estimates that the prevalence of obesity has almost tripled worldwide since 1975. There are currently more than 1.9-billion overweight adults, of which more than 650 million are obese. In 2016, 39% of adults aged 18 years and over were overweight and 13% were obese [1]. Combined therapies of hygienic-dietary measures, together with pharmacological treatments, can reduce body weight by 10% in the medium term, but in many cases, they fail in the long term [2]. The only treatment that has demonstrated its long-term effectiveness has been bariatric surgery (BC), which has shown a reduction in morbidity and mortality. It has also improved quality of life while being a cost-effective treatment [3]. The most widely used technique worldwide is a gastric bypass. However, in recent years, a progressive increase in the use of restrictive techniques has been detected, especially at the expense of tubular gastrectomy [4].

Bariatric procedures change the anatomy and physiology of the gastrointestinal system. Hence, they have an impact on diet quality, digestion, the absorption of food, and nutritional status [5,6]. Following surgery, the volume of food consumed and, hence, the energy intake is significantly reduced [5]. However, diet quality can be compromised, particularly if gastrointestinal symptoms are experienced from ingesting certain foods. While the gastrointestinal system adapts over time, and increased food intake has been reported [7,8], the malabsorption of nutrients may persist due to anatomical changes. In general, the restrictions in diet quality, as well as food intolerances, are a problem in the first year following surgery. A balanced diet is expected to be maintained by most patients after this period of adjustment. Some foods may continue to present challenges, even in the longer term. These food intolerances may contribute to the avoidance of food groups, and this in turn may impact diet quality [9,10]. The extent of dietary change and associated nutritional consequences varies between procedures [11].

Immediately after the surgical intervention, the dietary objective focuses on preventing complications, ensuring adequate tolerance to the diet, and avoiding nutritional deficiencies. It should be considered that the surgical technique influences the speed with which this progression can be established. The volume of food in each feeding, and even the type of food best tolerated, will also depend on the degree of gastric restriction or malabsorption produced by the surgery. Therefore, in the first weeks after the intervention, in which gastric restriction does not allow for the inclusion of foods with a high protein load, the use of artificial nutritional supplements may be necessary [12]. After the bariatric procedure, the introduction of a liquid diet at the hospital (protein supply between 40–60 g/day) is recommended. The patient should then be discharged after 1–2 weeks, and then a crushed or semi-soft diet should be introduced for 2 weeks (level of evidence is a 4, grade of recommendation is a C) [13]. Progress should be made with 6–8 small doses of a crushed diet based on purees (120–150 mL) distributed throughout the day [14]. This progressive diet aims for the patient to adapt to the capacity of the new gastric reservoir without experiencing digestive discomfort. The patients can start with a solid diet that provides between 60–120 g of protein per day to maintain lean mass during weight loss from 4 weeks post-surgery and prevent protein malnutrition [13,15,16].

Protein intake has a significant impact on body composition as it affects fat-free mass and has a metabolic effect by reducing plasma glucose and triglyceride levels after BC. Therefore, it is essential to consider the possible problems of patients to achieve the recommended protein intake without causing an imbalance of other macronutrients, which, in the long term, can affect bone homeostasis and mineralization. For these reasons, the use of protein supplements that contain all the essential amino acids to reach the daily consumption goal is advised if the recommended minimum is not reached. Preferably, these supplements replace natural foods with a commercial formula rich in protein [12].

There is a substantial prevalence of excessive lean body mass (LBM) loss in BS patients. Within the first year after a laparoscopic Roux-en-Y gastric bypass (RYGB), patients lose about 22% of their LBM [17]. There is a paucity of data that shows the correlation between protein intake and LBM loss after BS. In 2017, a systematic review concluded that two of the four studies with an adequate protein intake (≥60 g/day) were associated with significantly less LBM loss one year after RYGB [18,19].

The main objective of the study was to determine the efficacy of a specific oral nutritional supplement in body weight loss and body composition compared to the common clinical practice, which does not use any supplementation, in patients after sleeve gastrectomy.

## 2. Materials and Methods

### 2.1. Study Design

A cross-sectional study with two groups was developed in 44 patients diagnosed with obesity the month after bariatric surgery (sleeve gastrectomy). The patients were recruited in the Clinical Nutrition Unit of Clinic University Hospital of Valladolid between April 2020 and December 2022. After signing informed consent, patients were interviewed about medical history, disease progression, and nutritional anamnesis. Anthropometry, body composition, and biochemical analyses were examined in the two visits (at the beginning and the month after). The study was approved by the ethics committee of the East Valladolid Area with code PI 20-1710 and was conducted according to the principles of the Helsinki Declaration.

### 2.2. Study Subject

The selected patients had the following inclusion criteria: adult patients diagnosed with obesity (BMI ≥ 30 kg/m^2^) on the month after bariatric surgery (sleeve gastrectomy) between 18 and 65 years. The exclusion criteria included the other types of bariatric surgery (gastric bypass, adjusted gastric band, malabsorptive technics, etc.), endoscopy techniques for weight loss (intragastric balloon, APOLLO, endo-sleeve, etc.), several nutritional deficits that required nutritional treatment by parenteral route, and patients who did not sign informed consent.

### 2.3. Nutritional Intervention

In this design, we had two groups: low-protein, low-calorie diet (LP), which received a liquid and semiliquid diet during the first 4 weeks using normal food per the usual clinical practice; and high-protein, low-calorie diet (HP) (Table 1), which received a liquid and semiliquid diet during the first 4 weeks after the surgery that was complemented with 600 mL per day of an oral nutritional supplement (ONS) [Bi1 Bificare^®^, Adventia Pharma, Las Palmas de Gran Canaria, Spain) (Table 2). The person who administered the nutritional education was a registered dietitian who instructed in the management of bariatric surgery.

### 2.4. Variables

#### 2.4.1. Anthropometry

The anthropometric variables measured were weight (kg), height (m), and waist and hip circumferences. We calculated body mass index (BMI) as weight/height × height (kg/m^2^); percentage of weight loss (%TWL) as (presurgery weight (kg) compared to actual weight (kg))/(Usual weight (kg) × 100); percentage of excess of weight loss (%EWL) as presurgery weight compared to actual weight/the presurgery weight compared to the ideal adjusted weight × 100; where ideal adjusted weight (weight at BMI 25 kg/m^2^ + (0.25 × (presurgery weight compared to weight at BMI 25 kg/m^2^))) was measured using waist and hip circumferences. The health professional who administered anthropometry was a registered dietitian who specialized in anthropometric measurement and had skills in anthropometry and nutritional assessment. The measurements were always conducted by the same person.

#### 2.4.2. Body Composition

Bioelectrical Impedanciometry (BIA) was used to determined body composition. The BIA determines the hydration and cell density of the body by the determination of electric parameters such as reactance, reactance, and phase angle. The utility of validated estimative equations allows us to define the compartments of body composition [20]. Bioimpedanciometry (BIA NutriLab; EFG Akern, Akern, Pisa, Italy) was completed between 8:00 and 9:15 after an overnight fast and following a time of 15 min in the supine position. The BIA estimated the parameters of impedance (Z), resistance (R), and capacitance (X). The phase angle (PhA) is calculated with PhA = ((X/R) × 180°/π). It was calculated by estimative equation fat mass (FM), fat-free mass (FFM), fat-free mass (FFM), and the percentage of skeletal muscle mass (%MM) [20]. We calculated the appendicular skeletal muscle mass (ASMM) by Sergi Formula: −3.964 + (0.227 × RI) + (0.095 × weight) + (1.384 × sex) + (0.064 × Z), with an RI resistivity index (sex: Male = 1; Female = 0) [21]. We standardized fat-free mass and skeletal muscle mass by square height (height^2^). We defined them as fat-free mass index (FFMI) and appendicular skeletal muscle mass index (ASMI) with units kg/m^2^. These variables were applied as percentile curves of the Bodygram^®^ program developed by Akern for ages between 18 and 98 years.

### 2.5. Statistical Analysis

The database has been recorded with permission of the National Data Protection Agency. The collected data were saved in a database using the statistical software SPSS 23.0 (SPSS Inc., Chicago, IL, USA).

Qualitative variables were expressed as percentages and analyzed using the Chi-square test, with Fisher and Yates adjustments when necessary. Continuous variables were shown as the mean and standard deviation, while parametric variables were analyzed using the unpaired Student’s *t*-test. For non-parametric variables, tests such as Friedman, Wilcoxon, and the Mann-Whitney U test were used. The ANOVA U test was applied with the Bonferroni post-hoc test to compare variables in more than two groups. The analysis of the variables at different times of the study was conducted using the multivariate analysis of variance (MANOVA). A *p*-value of less than 0.05 was considered significant.

## 3. Results

**Figure 1:**
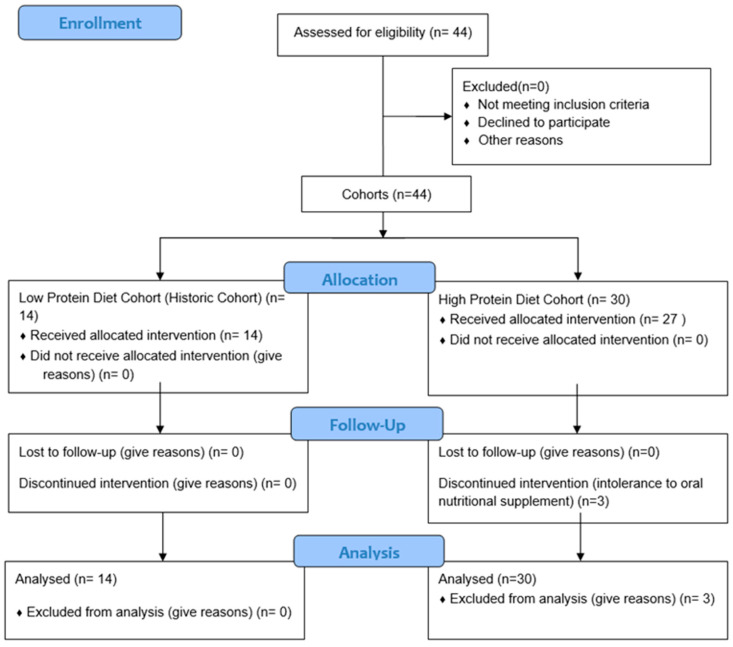
Flow chart.

Forty-four patients were recruited, and none of the patients was excluded. Thirty patients were allocated to the intervention group (high-protein diet), while 14 patients were allocated in the historic cohort (low protein diet). Three patients in the intervention group discontinued intervention due to an intolerance to the oral nutritional supplement (Figure 1).

### 3.1. Sample Description

The age was 47.35 (10.22) years, and 75% of patients were women. The weight was 123.14 (18.89) kg, and the BMI was 45.98 (6.13) kg/m^2^. There were no differences in sex, age, anthropometry variables, and bioimpedance variables between groups before intervention (Table 3). The differences between sex are shown in Table 4.

### 3.2. Differences in Anthropometric Parameters

The loss of weight of the total sample was 11.25 (7.07)% (men: 14.55 (7.39) kg; women: 10.12 (6.70) kg; *p* = 0.11). There were a significative loss of weight and BMI in both groups (Table 5).

The percentage of weight loss 1 month after bariatric surgery was 8.95% (5.22%) without differences between groups (high-protein diet: 9.65% (5.15%); low-protein diet: 7.51% (5.25%); *p* = 0.21). The percentage of excess weight loss was 20.63% (12.86%) without differences between groups (high-protein diet: 21.86% (12.60%); low-protein diet: 18.10% (13.49%); *p* = 0.38).

### 3.3. Differences in Bioimpedance Parameters

One month after bariatric surgery, an increase in resistance (7.69% [−1.9–18.67%]) and reactance (8.51% [−12.06–16.67%]) was observed. The body composition parameters estimated by BIA showed a decrease that was more marked in the fat index (−11.30% [11.13%]) than the fat-free mass index (−5.68% [8.08%]), appendicular skeletal mass index (−7.28% [8.31%]), or total body water (−4.67% [8.08%]).

When we compared both intervention groups, a significant increase in the resistance and reactance in the low-protein-diet group was observed, while a significant decrease in ASMI, FFMI, FI, and TBW in the low-protein group was observed (Table 4). In the high-protein group, a significant decrease in ASMI, FFMI, and FI was observed without differences in the electrical parameters from bioimpedanciometry (Table 4).

**Figure 2:**
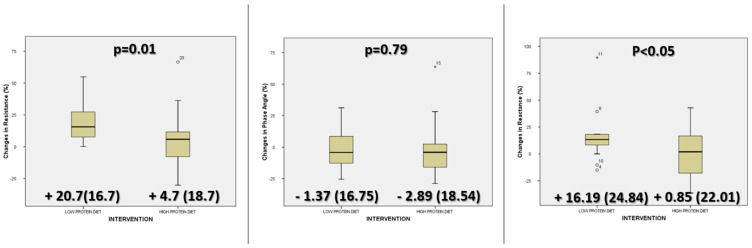
Comparison between groups (high-protein diet and low-protein diet) of changes in electrical bioimpedanciometry variables (resistance, reactance, and phase angle) before, and one month after, bariatric surgery.

The percentage of change between bioimpedance parameters showed a higher increase in resistance in the low-protein diet group without differences between parameters in the phase angle and reactance (Figure 2).

**Figure 3:**
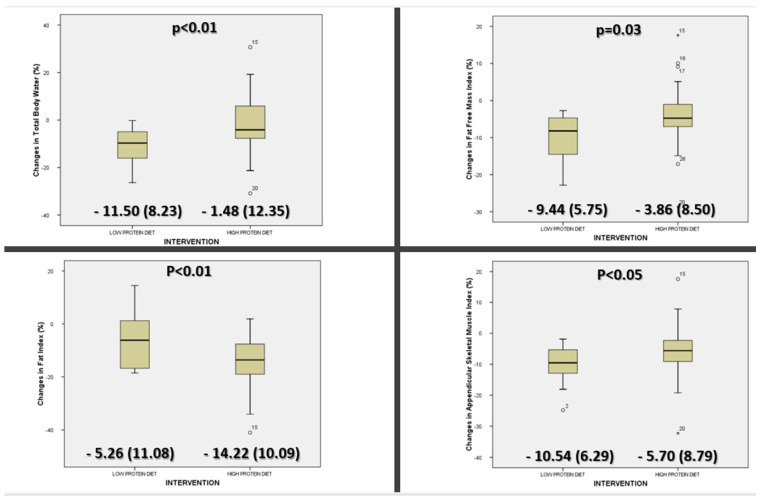
Comparison between groups (high-protein diet and low-protein diet) of changes in body composition compartments determined by bioimpedanciometry (total body water, fat-free mass index, fat index, and appendicular skeletal muscle index) before and 1 month after bariatric surgery.

The change in estimated compartments of body composition showed a higher decrease in TBW, FFMI, and ASMI in the low-protein diet group. On the other hand, a higher decrease in FI was observed in the high-protein diet group (Figure 3).

## 4. Discussion

In patients undergoing sleeve gastrectomy, those patients who consumed a modified diet with a normocaloric, hyperproteic oral nutritional supplement to complete their calorie-protein requirements (HP) showed differences in body composition. This was determined by bioimpedanciometry with respect to those who consumed a standard liquid-semiliquid progression diet (LP). A similar decrease in body weight was observed in both groups. Nevertheless, the patients who received an HP diet showed a lower decrease in the fat-free mass index and the appendicular skeletal mass index, as well as a higher decrease in the fat index, compared to those who consumed an LP diet.

The baseline parameters of our sample did not differ between groups, and these patients have similar values to other studies regarding the fat mass index and the fat-free mass index. One study from Molero et al. observed a decrease in the skeletal muscle mass index in patients prior to bariatric surgery with a mean value of 9.5 (1.5) kg/m^2^, as we have seen in our study [22]. This low muscle mass can predict a higher loss of muscle in these patients related to the type of surgery, as well as the lifestyle of patients. However, the use of an adequate education can lead patients to maintain fat-free mass and skeletal muscle mass, or even increase it compared to control groups [23].

Patients who underwent bariatric surgery show changes in anthropometry and body composition since the earliest phases after intervention. Maïmoun et al. observed that patients undergoing sleeve gastrectomy lose 8.6% (1.8%) weight and 22.36% (6.64%) excess weight in the first month after intervention [24]. The body composition is also affected by this circumstance, while the previous study showed that these patients lose more lean mass (9.7% [3.3%]) than fat mass (−7.7% [4.3%]) [24]. We observed in our sample different results, as we have seen a more marked reduction of the fat index than the fat-free mass index. This condition must be related to a lower decrease of the total body water in the first month after surgery on the whole sample. However, the determination of body composition in patients undergoing bariatric surgery can be influenced by the use of impedanciometry. In a study from Carrasco et al., they observed an underestimation of weight loss as fat mass and an overestimation of fat-free mass loss [25]. This study was conducted in patients with a Roux-Y gastric bypass, and the results cannot be directly compared. However, we must be careful in comparing the studies as we must consider the technique of body composition.

One of the ingredients included in the oral nutritional supplement was a postbiotic (bifidobacterium animalis subsp. lactis CECT 8145, heat killed), which has demonstrated a positive impact in the reduction of visceral fat, waist circumference, and the waist circumference/height ratio in obese subjects after the ingestion of 10-billion 1010 CFU for 12 weeks [26]. Although, in our study, the same anthropometric (waist circumference and waist circumference/height) and body composition (visceral fat) determinations were not made and the duration of the intervention was the same (2 weeks instead of 12 weeks), the use of this postbiotic could have an influence in the reduction of the total fat mass.

Sleeve gastrectomy produces an important loss of weight in the first months after surgery [27,28], but these changes often stabilize during the first year after surgery compared to the Roux-Y gastric bypass [29]. The changes in body composition are also similar to malabsorptive techniques. Nevertheless, some studies see more marked changes in body composition in the first months after bariatric surgery, especially in fat-free mass index [27]. Others, such as Pakzad’s study, did not observe differences on these parameters between the different types of surgery [30].

Calorie-protein consumption decreases after bariatric surgery. In a study from Abdulsalam et al., they observed a decrease in protein consumption of 52.36 (25.04) kg/m^2^ 3 months after sleeve gastrectomy [30], which was maintained in the first year after surgery. Another study from Golzarand et al. showed a decrease in protein intake from 111(56) g/day to 36.7(12.5) g/day 6 months after surgery. This protein intake is below the recommendation of 60 g/day mentioned in most clinical guidelines [13]. In our sample, the protein consumption in the control group with a standard progression diet showed a very low protein content (11.70 g/day) the first 2 weeks after surgery. This content increased to 65.96 g/day over the next 2 weeks in the first month. Sleeve gastrectomy leads us to a more intensive restriction in protein that is maintained in the first 12 months. In the study from Abdulsalam, it showed less protein content in the diet in patients with sleeve gastrectomy compared to the Roux-Y gastric bypass [31].

In the early postoperative period, the use of a very low-protein diet can lead to a more marked deterioration of body composition. Diets that did not meet the recommended requirements have shown a non-significant decrease in the percentage of the fat-free mass of weight loss in patients with a higher protein consumption, but the maintenance of this quantity after 6 months is related to a better evolution of the fat-free mass percentage [27]. The acute changes in body composition are related to diet but also are related to decreased physical activity and proinflammatory state after surgery.

There are many studies that evaluate the use of low-calorie, high-protein diets in the period before bariatric surgery period, but studies that evaluate the use of oral nutritional supplements after surgery are scarce. A study from Alshamari et al. compared the effectiveness of a protein supplement with 250 kcal and 20 g protein in 200 mL with a placebo supplement with carbohydrates. There were neither differences in body composition nor weight loss at 1, 3, or 6 months, but there was an increase in total plasma protein in the study group [32]. In our study, we use a similar oral nutritional supplement (200 kcal, 18 g protein in 200 mL), but the intake regimen was different (three bottles per day in an incomplete oral natural diet). It was enriched in leucine (3 g in 200 mL), and the period of use of this dietary pattern was for 2 weeks and not maintained in time. In our study, we observed no differences in weight loss, but we have seen a higher decrease in fat mass in the intervention group and a higher decrease in the fat-free mass index and appendicular muscle mass index in the control group. It is true that the control group has an intensive restriction in protein intake due to recommendations related to diet tolerance. However, the differences in fat-free mass index can be related to the higher decrease of total body water in patients within the control group.

Another study from Hirsch et al. compared the use of a ready-to-drink protein formula with 30 g of protein, 4–5 g of carbohydrates, and 3 g of fat in one serving a day for 12 weeks. The investigators observed that patients more easily achieve the recommended protein intakes, but there were no significant differences in body composition. It was observed a further, non-significant decrease in the percentage of body fat in the experimental group, and a non-significant, further decrease in the fat-free mass index in the control group [33]. These findings are like those observed in our study. Nevertheless, our study had another dietary pattern: an oral nutritional supplement regime.

Bariatric surgery is associated with a decrease in the levels of branched chained aminoacids, such as leucine, valine, and isoleucine compared with levels before the surgery [34]. The use of leu-cine-enriched formulas has not been well-studied in patients after bariatric surgery, but there are some studies that show the effect of this aminoacid on the preservation of muscle mass in patients with disease-related malnutrition and sarcopenia [35]. Although this evidence is weak, the use of this type of aminoacids could have a potential benefit over muscle mass due to a better tolerance and a high biodisponibility. Therefore, our data can be used for future intervention studies in this topic.

The main strength of our study is the evaluation of dietary progression in the first month after surgery. Usually, the evaluation of diet is made long term in patients after bariatric surgery, and its influence at 1 year or more after surgery. Another strength is the evaluation of body composition at the first month after surgery. The determinations of body composition are usually made 3 months after bariatric surgery.

The main limitation of our study is the small sample size, as this is a pilot study to evaluate the effectiveness of the use of a modified hyperproteic diet. Another reason for the small sample size is the control group; we needed to use a historic cohort because the use of a hypoproteic diet is not recommended in these patients. Another limitation is the use of estimative formulas of bioimpedanciometry. This allows for the easy estimation of changes in body composition without using more complex diagnostic probes such as DEXA or TAC/MRI, but it can underestimate or overestimate real values of body composition. Nevertheless, using the same impedanciometry method to monitor the results, we can evaluate changes in body compartments.

## 5. Conclusions

In patients undergoing gastric sleeve surgery, the use of a progression diet with the addition of a normocaloric, hyperproteic formula managed to slow down the loss of muscle mass and increase the loss of fat mass with no differences on the total weight loss. The use of protein supplements is recommended in patients with restrictive diets after bariatric surgery to achieve the protein requirements and reduce the damage over the muscle mass caused by important weight loss in these patients.

These studies must be developed with more accurate variables to determine body composition and energy-protein requirements. Scientific evidence of real protein requirements in these patients is needed. The 60 g/day value cannot be used for all patients as these requirements are different depending on sex, race, age, height, and body composition before surgery. Another possible study is the comparison of the use of different types of protein in diets with different bioavailability and biological value, and its relationship with physical activity.

## Figures and Tables

**Table 1:** Description of the nutritional intervention between groups.

	Low-Protein Diet		High-Protein Diet	
	First 14 Days	Last 14 Days	First 14 Days	Last 14 Days
Energy (kcal)	217	1155.5	817	1155.5
Protein (g)	11.70	65.95	65.70	65.95
Carbohydrate (g)	37.40	164.45	97.40	164.45
Fat (g)	1.73	22.12	14.33	22.12
Fibre	2.32	17.39	17.32	17.39

**Table 2:** Nutritional composition of the oral nutritional supplement: Bi1 Bificare^®^ (Adventia Pharma, Spain).

	100 mL	600 mL
Energy (kcal)	100	600
Proteins: g/TE%	9.0 g/36%	54.0 g/36%
Carbohydrates: g/TE% Sugars: g	10 g/40% 1.1 g	60 g/40% 6.6 g
Fat g/TE%	2.1 g/19%	12.6 g/19%
Saturated (TE%)	4%	4%
MUFA (TE%)	10%	10%
PUFA (TE%)	4.7%	4.7%
• EPA & DHA (mg)	170 mg	1220 mg
Fibre g/soluble-insoluble	2.5 g/70–30%	15 g/70–30%
BPL-1	1.67^10^ CFU	10^10^ CFU

BPL-1: bifidobacterium animalis subsp. lactis CECT 8145, heat killed; CFU: colony-forming units; DHA: docosahexaenoic acid; EPA: eicosapentaenoic acid; MUFA: monounsaturated fatty acid; PUFA: polyunsaturated fatty acid; TE%: percentage of total energy.

**Table 3:** Differences of variables before bariatric surgery between patients with high-protein diet and those with low-protein diet after surgery.

	High-Protein Diet	Low-Protein Diet	*p*-Value
Sex (Men/Women)%	63.6/36.4	69.7/30.3	0.71
Age (years)	47.63 (9.96)	46.43 (11.09)	0.72
BMI (kg/m^2^)	46.46 (5.22)	44.95 (7.87)	0.45
Resistance (ohm)	436.20 (69.58)	403.57 (57.87)	0.13
Reactance (ohm)	46.90 (8.90)	43.79 (7.27)	0.26
Phase Angle (°)	6.16 (0.82)	6.28 (0.66)	0.65
ASMI (kg/m^2^)	9.79 (0.91)	9.96 (1.35)	0.63
FFMI (kg/m^2^)	22.29 (2.21)	22.63 (2.88)	0.67
FI (kg/m^2^)	24.17 (4.44)	22.32 (5.59)	0.24
TBW (%)	39.33 (5.15)	42.76 (5.33)	0.06

ASMI: Appendicular Skeletal Mass Index; FFMI: Fat-Free Mass Index; FI: Fat Index; TBW: Total Body Water.

**Table 4:** Differences of variables before bariatric surgery between patients with high-protein diet and those with low-protein diet after surgery in both sexes.

	High-Protein Diet		Low-Protein Diet	
	Men (*n* = 7)	Women (*n* = 23)	Men (*n* = 4)	Women (*n* = 10)
Sex (%)	36.4	63.6	30.3	69.7
Age (years)	51.57 (7.41)	46.43 (10.46)	50.25 (5.73)	44.9 (12.56)
BMI (kg/m^2^)	45.71 (4.77)	46.69 (5.43)	40.64 (6.44)	46.67 (8.00)
Resistance (ohm)	411 (51.11)	443.87 (73.53)	378.5 (33.52)	413.6 (63.81)
Reactance (ohm)	43.71 (8.88)	47.87 (8.87)	41.25 (2.75)	44.80 (8.35)
Phase Angle (°)	6.1 (1.12)	6.18 (0.73)	6.5 (0,22)	6.19 (0.77)
ASMI (kg/m^2^)	9.55 (0.94)	9.87 (0.91)	9.47 (1.03)	10.16 (1.45)
FFMI (kg/m^2^)	20.43 (1.98)	22.86 (1.98) *	20.38 (1.96)	23.53 (2.76)
FI (kg/m^2^)	25.28 (3.90)	23.83 (4.61)	20.25 (4.76)	23.14 (5.90)
TBW (%)	41.06 (4.52)	38.80 (5.31)	47.75 (4.68)	40.76 (4.26) *

ASMI: Appendicular Skeletal Mass Index; FFMI: Fat-Free Mass Index; FI: Fat Index; TBW: Total Body Water. * *p*-value < 0.05.

**Table 5:** Changes in variables before and one month after bariatric surgery between groups.

	High-Protein Diet			Low-Protein Diet		
	Prebariatric	One Month Postbariatric	*p*-Value	Prebariatric	One Month Postbariatric	*p*-Value
Weight (kg)	123.89 (17.13)	111.81 (16.27)	<0.01	122.24 (23.24)	112.69 (19.61)	<0.01
BMI (kg/m^2^)	46.39 (5.29)	41.94 (5.67)	<0.01	44.95 (7.87)	41.49 (7.01)	<0.01
Resistance (ohm)	436.20 (69.58)	451.63 (80.04)	0.26	403.57 (57.87)	481.19 (66.22)	<0.01
Reactance (ohm)	46.90 (8.90)	46.30 (9.38)	0.76	43.79 (7.27)	50.30 (10.04)	<0.02
Phase Angle (°)	6.16 (0.82)	5.94 (1.26)	0.32	6.28 (0.66)	6.13 (0.83)	0.60
ASMI (kg/m^2^)	9.80 (0.93)	9.23 (1.13)	<0.01	9.96 (1.35)	8.89 (1.21)	<0.01
FFMI (kg/m^2^)	22.27 (2.24)	21.36 (2.45)	0.01	22.63 (2.88)	20.46 (2.69)	<0.01
FI (kg/m^2^)	24.12 (4.50)	20.58 (4.29)	0.01	22.32 (5.58)	21.04 (5.03)	<0.01
TBW (%)	39.33 (5.16)	38.41 (4.55)	0.34	42.76 (5.33)	37.74 (5.44)	<0.01

ASMI: Appendicular Skeletal Mass Index; FFMI: Fat-Free Mass Index; FI: Fat Index; TBW: Total Body Water.

## Data Availability

The data presented in this study are available on request from the corresponding author. The data are not publicy available due to ethical and privacy restrictions.
